# Crystal structure of higher plant heme oxygenase-1 and its mechanism of interaction with ferredoxin

**DOI:** 10.1074/jbc.RA120.016271

**Published:** 2020-12-24

**Authors:** Rei Tohda, Hideaki Tanaka, Risa Mutoh, Xuhong Zhang, Young-Ho Lee, Tsuyoshi Konuma, Takahisa Ikegami, Catharina T. Migita, Genji Kurisu

**Affiliations:** 1 Institute for Protein Research, Osaka University, Suita, Osaka, Japan; 2 Department of Macromolecular Science, Osaka University, Toyonaka, Osaka, Japan; 3 Graduate School of Medical Science, Yamagata University, Yamagata, Yamagata, Japan; 4 Research Center of Bioconvergence Analysis, Korea Basic Science Institute, Cheongju, Chungbuk, South Korea; 5 Graduate School of Analytical Science and Technology, Chungnam National University, Yuseong-gu, Daejeon, South Korea; 6 Research Headquarters, Korea Brain Research Institute, Dong-gu, Daegu, South Korea; 7 Bio-Analytical Science, University of Science and Technology, Yuseong-gu, Daejeon, South Korea; 8 Graduate School of Medical Life Science, Yokohama City University, Tsurumi-ku, Yokohama, Japan; 9 Department of Biological Chemistry, Yamaguchi University, Yoshida, Yamaguchi, Japan

**Keywords:** X-ray crystallography, structural biology, ITC, NMR spectroscopy, heme oxygenase, CO, carbon monoxide, CPR, cytochrome P450 reductase, DSC, differential scanning calorimetry, Fd, ferredoxin, Fe–SAD, iron single-wavelength anomalous dispersion, FNR, Fd–NADP+ reductase, GmHO-1, *Glycine max* (soybean) HO-1, hHO-1, human HO isozyme1, HO, heme oxygenase, rHO-1, rat HO isozyme1, SynHO-1, *Synechocystis* sp PCC6803. isozyme1

## Abstract

Heme oxygenase (HO) converts heme to carbon monoxide, biliverdin, and free iron, products that are essential in cellular redox signaling and iron recycling. In higher plants, HO is also involved in the biosynthesis of photoreceptor pigment precursors. Despite many common enzymatic reactions, the amino acid sequence identity between plant-type and other HOs is exceptionally low (∼19.5%), and amino acids that are catalytically important in mammalian HO are not conserved in plant-type HOs. Structural characterization of plant-type HO is limited to spectroscopic characterization by electron spin resonance, and it remains unclear how the structure of plant-type HO differs from that of other HOs. Here, we have solved the crystal structure of *Glycine max* (soybean) HO-1 (GmHO-1) at a resolution of 1.06 Å and carried out the isothermal titration calorimetry measurements and NMR spectroscopic studies of its interaction with ferredoxin, the plant-specific electron donor. The high-resolution X-ray structure of GmHO-1 reveals several novel structural components: an additional irregularly structured region, a new water tunnel from the active site to the surface, and a hydrogen-bonding network unique to plant-type HOs. Structurally important features in other HOs, such as His ligation to the bound heme, are conserved in GmHO-1. Based on combined data from X-ray crystallography, isothermal titration calorimetry, and NMR measurements, we propose the evolutionary fine-tuning of plant-type HOs for ferredoxin dependency in order to allow adaptation to dynamic pH changes on the stroma side of the thylakoid membrane in chloroplast without losing enzymatic activity under conditions of fluctuating light.

Heme oxygenation is one of the key metabolic reactions catalyzed by heme oxygenase (HO), which was first identified in 1968 as an enzyme catalyzing the oxidative cleavage of heme in mammalian microsomes (1, 2). Subsequently, kinetic analyses of human HO isozyme1 (hHO-1) showed that the sequential enzymatic breakdown of heme to biliverdin consists of seven steps (Fig. 1*A*). First, using molecular oxygen and two electrons, heme is oxidized in four steps to α-*meso*-hydroxyheme, which is further converted to verdoheme and carbon monoxide (CO) after reacting with another molecular oxygen and one electron. Verdoheme subsequently undergoes ring opening and is converted in two steps to biliverdin and free iron, consuming another molecular oxygen and four electrons (3, 4, 5). Throughout all reactions, the resultant products are CO, biliverdin, and free iron, all of which play crucial roles in cellular redox signaling, formation of anti-inflammatory and antioxidant molecules, and recycling of iron (6). Owing to the importance of these products, the initial studies on HOs focused on mammalian enzymes.

**Figure 1:**
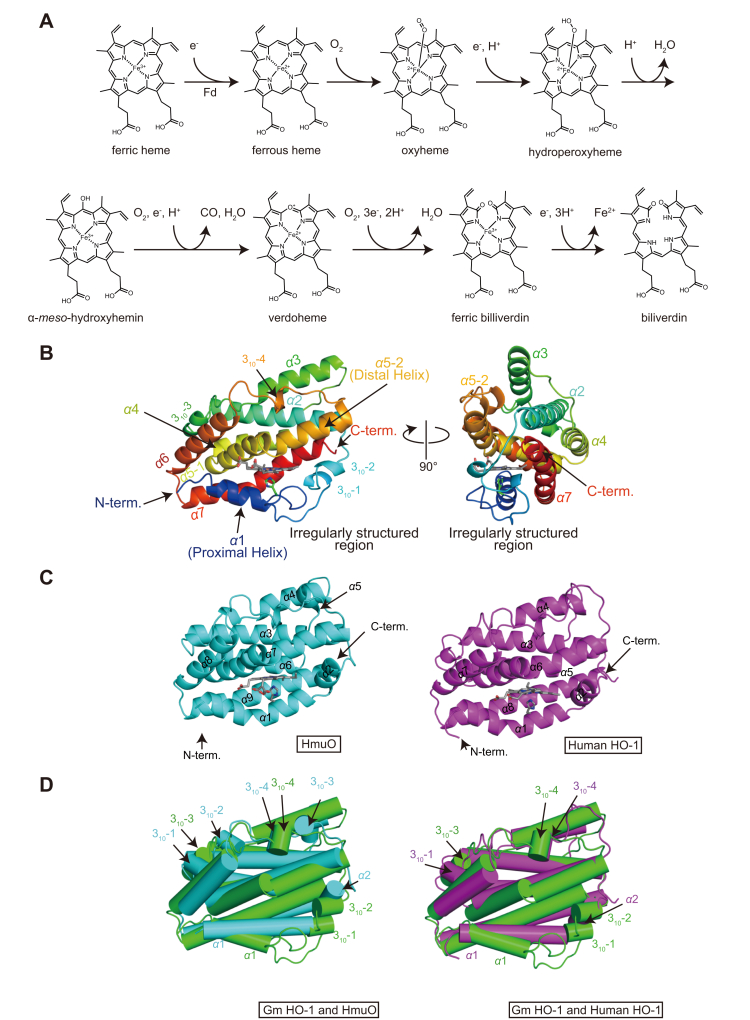
**Overall structure of heme–GmHO-1 and structural comparison with other HOs.***A*, Schematic of the degradation of heme by HO-1. *B*, Ribbon model structure of heme–GmHO-1 colored in rainbow along each helical region from the N-terminus (*blue*) to the C-terminus (*red*). The proximal ligand His30 and heme are colored in *green* and *gray*, respectively. *C*, Structure of HmuO (PDB ID: 1iw0, *cyan*) and hHO-1 (PDB ID: 1n45, *magenta*). Heme, which is sandwiched between the proximal and distal helices, and the proximal ligand His are colored in *gray* in each structure. *D*, Structural comparison of higher plant HO-1 with other HOs. The heme–GmHO-1 structure is colored in *green* and overlapped with the structure of HmuO (*cyan*) or hHO-1 (*magenta*). Although the structural topology of HO is highly conserved, there are several differences. In the HmuO and hHO-1 structures, α2 is located on the C-terminal side of the proximal helix. By contrast, heme–GmHO-1 has two 3_10_ helices at this position. All figures in this paper were drawn by PyMol (https://pymol.org/2/).

However, HOs are widely distributed in many organisms, including bacteria, cyanobacteria, algae, and plants (7, 8, 9, 10, 11, 12, 13, 14, 15). Specifically in photosynthetic organisms such as cyanobacteria and higher plants, HO plays a physiological role in producing precursors of the photoreceptor pigments: phycocyanobilin, phytochromobilin, and others (9, 11, 16, 17, 18). Although the enzymatic reactions carried out by bacterial, mammalian, and photosynthetic HOs are the same (19, 20), there is a difference in the electron donor required for the reactions, that is, ferredoxin (Fd) is used by photosynthetic HOs, whereas NADPH–cytochrome P450 reductase (CPR) is used by mammalian HOs (5, 21).

In the early 2000s, crystal structures of hHO-1, rat HO isozyme1 (rHO-1), *Corynebacterium diphtheriae* HmuO, *Pseudomonas aeruginosa* PigA, and cyanobacterial HOs from *Synechocystis* sp. PCC 6803 (SynHO-1 and SynHO-2) were reported one after another (22). Furthermore, the crystal structure of the CPR in complex with rHO-1 was also reported at a resolution of 4.3 and 3.25 Å (23, 24) to indicate the path of electron transfer from CPR to the active site of rHO-1. In all the aforementioned structures, HO possesses an α-helical structure, in which heme is sandwiched between proximal (α1) and distal (α6 or α7) helices with axial ligation from a conserved His residue (22, 25, 26, 27, 28, 29, 30). The architectures of HOs resembled one other, implying a common mechanism of enzymatic reaction except for the electron donor of Fd in cyanobacterial HO.

Among HOs with amino acid sequences available in public databases, bacterial, mammalian, and cyanobacterial HOs show some amino acid sequence identity (*e.g.*, 32.9% between HmuO and hHO-1 and 42.5% between hHO-1 and SynHO-1). However, higher plant HO-1 is exceptional and shows relatively low sequence identity to other HOs; for example, *Glycine max* (soybean) HO-1 (GmHO-1) has only 19.5% identity to hHO-1 and 20.1% to SynHO-1. Thus, even though it has similar physiological functions, plant-type HO may be considered as an independent group, different from cyanobacterial HO because its 3D structural information is not yet available. In addition, based on the amino acid sequence alignment, GmHO-1 lacks some essential amino acid residues, including Asp140, which is a key residue in a hydrogen-bonding network for proton transfer, and His25, which coordinates with heme (hHO-1 numbering), although Gohya *et al.* (31) previously observed ligation of heme from His in the spectroscopic measurement of GmHO-1.

Given these observations, the heme-binding site of plant-type HO is thought to differ somewhat from those of other HOs. To date, however, there has been no high-resolution structure of plant-type HO, and only a prediction model is available (32). In addition, there are no structural data regarding how the photosynthetic HOs (plant-type and cyanobacterial HO) recognize Fd because the two types are expected to have distinct architecture from each other. In this study, we have solved the crystal structure of GmHO-1 with a bound heme molecule at a resolution of 1.06 Å and collected data on the interaction between GmHO-1 and heme/Fd by ITC and NMR to reveal the unique structural features of plant-type HO.

## Results

### Overall structure and comparison with other HOs

First, we tried to solve the crystal structure of GmHO-1 by the molecular replacement method using published HO structures as a search model, but this approach failed, implying a significant structural difference between plant-type and other HOs. We therefore adopted the iron single-wavelength anomalous dispersion (Fe-SAD) method for phase determination and successfully solved the structure with refinement finally up to a resolution of 1.06 Å using SHELXL (33) and COOT (34). The crystal data and crystallographic refinement statistics are given in Table 1. The final resolution was sufficiently high to clearly trace the whole polypeptide chain, except for the N-terminal 15 residues, which probably have high flexibility.

**Table 1:** Crystallographic data and refinement statistics

Data set	Native	Fe-SAD
Data collection		
X-ray source	SPring-8 BL44XU	TPS 05A
Wavelength (Å)	0.90000	1.73875
Space group	*P*2_1_	*P*2_1_
Unit-cell parameters		
(Å)	*a* = 37.7, *b* = 45.6, *c* = 72.4	*a* = 37.8, *b* = 45.6, *c* = 72.8
(°)	*β* =101.1	*β* = 101.0
Resolution (Å)	37.05–1.06 (1.13–1.06)	37.11–1.84 (1.91–1.84)
Completeness (%)	98.3 (97.1)	90.9 (74.1)
*R*_merge_ (%)^a^	5.1 (45.6)	4.8 (29.8)
〈*I/σ(I)*〉	9.78 (1.93)	19.4 (1.94)
Refinement		
*R*_work_^b^/*R*_free_^c^ (%)	15.94/17.29	
Ramachandran plot		
Favored (%)	98.65	
Allowed (%)	0.68	
Outliers (%)	0.68	
Average B for main chain (Å^2^)	13.942	
Average B for side chain and water (Å^2^)	21.631	
Average B for all atoms (Å^2^)	18.243	
rmsd, bonds (Å)	0.016	
rmsd, angles (°)	2.06	

SAD, single-wavelength anomalous dispersion.

Values in parentheses are for the highest resolution shell.

a *R*_merge_(*I*) = ∑|*I*(*k*) − ⟨I⟩|/∑*I*(*k*), where *I*(*k*) is the value of the kth measurement of the intensity of a reflection, ⟨*I*⟩ is the mean value of the intensity of that reflection, and the summation is the overall measurement.

b *R*_work_ = ∑||*F*_obs(hkl)_| − |*F*_calc(hkl)_||/∑|*F*_obs(hkl)_|.

c *R*_free_ is the R-factor computed for the test set of reflections that were omitted from the refinement process.

The GmHO-1 structure comprises four 3_10_ helices (residues 45–47, 50–52, 84–89, and 172–174) and eight α-helices designated as α1 (20–29), α2 (57–80), α3 (96–110), α4 (119–134), α5-1 (136–151), α5-2 (153–166), α6 (180–196), and α7 (200–224). Because the α5 helix is obviously bent, it is subdivided into two. New features of the GmHO-1 structure include the shorter α1 helix and an additional irregularly structured region continuing from α1 (Fig. 1*B*), which has not been observed in any other HO structures. To accommodate this novel irregularly structured region near the heme-binding site, the α7 helix (corresponding to α9 in HmuO or α8 in hHO-1) has become a curvilinear helix (Fig. 1*B*, *right panel*). The heme molecule bound to GmHO-1 is sandwiched between proximal (α1) and distal (α5) helices with axial ligation from His30 residue (Fig. 1*B*), consistent with previous data from electron paramagnetic resonance measurements (31).

**Figure 2:**
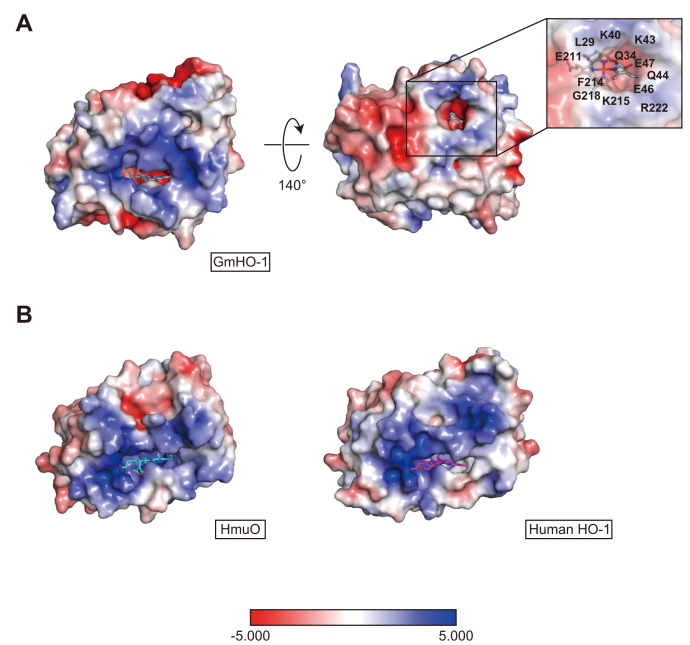
**Electrostatic potential map of HOs and magnified view of the new tunnel in *Glycine max* (soybean) HO-1 (GmHO-1).***A*, GmHO-1 and *B*, human HO isozyme1 (hHO-1) and HmuO. Negative electron potentials are drawn in *red* (−5 kT/e) and positive in *blue* (5 kT/e) at pH 7.0. Heme is shown as a *stick model* (GmHO-1, *gray*; HmuO, *cyan*; hHO-1, *magenta*). The *inset* shows an enlargement of the amino acid residues that form the new tunnel in GmHO-1. Some water molecules (not shown) were observed in this unexpected tunnel, which has negative electron potential. HmuO, a gene oxygenase of *Corynebacterium diphtheriae*; HO-1, heme oxygenase 1.

In the published structural reports of hHO-1 and HmuO, HO is composed of eight and nine α-helices (Fig. 1*C*), and the bound heme is sandwiched between proximal (α1) and distal (α7 in HmuO or α6 in hHO-1) helices (31, 35, 36). Superposing the structures of GmHO-1 with hHO-1 and HmuO clarifies similarities and differences between plant-type HO and other HOs (Fig. 1*D*). Similarity to HmuO exists in the overall architecture of GmHO-1, with an rmsd value of 2.28 Å to hHO-1 and 2.22 Å to HmuO, even though the amino acid sequence identity is relatively low (19.5% to hHO-1 and 19.6% to HmuO). Differences are seen in the arrangement of the 3_10_ helices, newly found irregularly structured region, and loop regions. Although GmHO-1, HmuO, and hHO-1 have four 3_10_ helices, two of the 3_10_ helices in GmHO-1 occupy the region corresponding to the α2 helix position in the other HOs. Because the C-terminal α7 helix is curved and becomes a curvilinear helix, the last half of this helix forms an unexpected tunnel (Fig. 2), together with the novel irregularly structured region. Among the loop structures connecting the α-helices in GmHO-1, a CD loop (37) connecting the α2 and α3 helices is the most significantly different from those of HmuO and hHO-1.

### Heme-binding sites and environment

**Figure 3:**
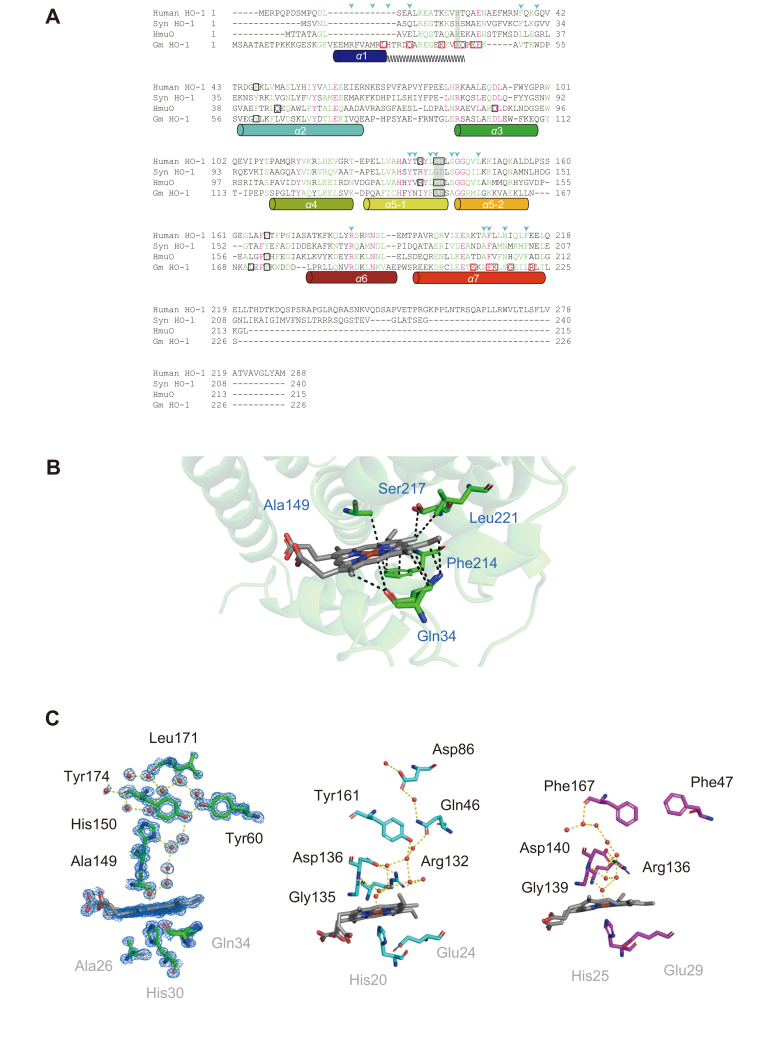
**Comparison with other HOs.***A*, amino acid sequence alignment of *Glycine max* (*soybean*) HO-1 (GmHO-1), hHO-1, Syn HO-1, and HmuO. *Colored cylinders* indicate the helical regions in GmHO-1. The *zigzag line* indicates the irregularly structured region. *Black boxes* highlight amino acid residues that form the hydrogen-bonding network in GmHO-1 (Fig. 2*C*). *Red boxes* highlight amino acid residues that form the unexpected tunnel. *Blue arrowheads* indicate amino acid residues that interact with heme. Residues in *red* are identical and those in *green* are similar. Key residues in heme ligation and the hydrogen-bonding network are highlighted with a *gray* background. *B*, position of heme in GmHO-1, showing interactions with amino acid residues that are not observed in other HO-1s (*blue arrowheads* in *A*). *C*, hydrogen-bonding network formed along the distal helix from the active center to the protein surface (GmHO-1, *green*; HmuO, *cyan*; hHO-1, *magenta*). *Red balls* indicate water molecules. Hydrogen bonding is indicated by a *yellow dotted line*. In GmHO-1, amino acid residues that form the hydrogen-bonding network and interact with His30 in the proximal helix are shown as a 2*F*_o_–*F*_c_ map contoured at *σ* = 2.0 (*blue mesh*). In the GmHO-1 structure, Ala149 and His150 in the distal helix are involved in the hydrogen-bond network. In other HOs, Arg and Asp residues (Arg132 and Asp136 in HmuO) form the basis of the hydrogen-bond network. Gln46, which contributes to the hydrogen-bond network in HmuO, is replaced with Phe47 in mammalian HO-1, indicating that the hydrogen-bond network is disconnected. HO-1, heme oxygenase 1.

Based on the amino acid sequence alignment, plant-type HO-1 lacks some key amino acid residues for heme-binding or catalytic reactions (*e.g.*, ligating His residue) that are conserved in other HOs (Fig. 3*A*). In our X-ray structure, the heme-binding sites of GmHO-1 are composed of 18 residues (Arg23, Met27, His30, Gln34, Val50, Trp53, Tyr144, Asn145, Phe148, Ala149, Ala152, Gly153, Ile157, Arg188, Ser213, Phe214, Ser217, and Leu221; *arrowheads* in Fig. 3*A*), five of which are not structurally conserved between GmHO-1 and other HOs (Fig. 3*B*). The heme iron is ligated by the proximal residue His30, which does not seem to be conserved in the alignment, but is structurally equivalent to His25 in hHO-1 and His20 in HmuO. Our X-ray structure reveals that spatial insertion of the newly identified irregularly structured region causes a 13-residue upstream shift of the ligating His residue in the alignment.

Propionate groups of the bound heme interact with Arg23, Tyr144, and Arg188 of GmHO-1, all of which are conserved in the alignment (Fig. 3*A*). Note that Arg23 is located seven residues upstream of His30, corresponding to Lys18 in rHO-1 and hHO-1, whereas Tyr144 and Arg188 correspond to Tyr134 and Arg183, respectively, in hHO-1 (26, 36, 38, 39, 40, 41). Electrostatic interactions between the propionate groups and two conserved basic residues assisted by tyrosine might be crucial to fix the orientation of the bound heme molecule consistently in all HOs.

Plant-type specific interactions with heme are mediated by five residues (Glu34, Ala149, Phe214, Ser217, and Leu221) (Fig. 3*B*), all of which make hydrophobic contacts with the heme molecule. These five residues are located near the newly identified structural features of the irregularly structured region and curved α7 helix and probably avert conflict between heme binding and these novel structural features. Other structural features unique to the plant-type HO are observed in the heme environment. On the distal side of heme, the distance between the α-*meso* carbon of heme and an adjacent water molecule is 3.4 Å, which is shorter than that in other HOs (*e.g.*, 3.9 Å in HmuO and 4.4 Å in hHO-1) and consistent with the previous report of Gohya *et al.* (42). This observation suggests that the adjacent water molecule may also contribute to stabilize the bound heme (Fig. 3*C*).

The amino acid sequence alignment indicates that the plant-type HO lacks two key residues: an Arg residue (Arg132 in HmuO and Arg136 in hHO-1) and an Asp residue (Asp136 in HmuO and Asp140 in hHO-1); these residues form part of the hydrogen-bonding network from the bound heme through a water molecule on the distal side and are thought to facilitate catalytic activity by stabilizing the unstable intermediate of the heme oxygenation process (28, 31, 43). In our X-ray structure of GmHO-1, Ala149 and His150 in the distal helix instead are involved in forming the equivalent hydrogen-bond network from the solvent to the active site *via* Tyr174, Leu171, and Tyr60 (31, 43) (Fig. 3*C*). On the proximal side, the imidazole ring of the ligating His30 residue, which is rotated approximately 160°, and the δ-nitrogen form a new hydrogen bond to the carbonyl oxygen of Ala26, whereas the δ-nitrogen of the other HOs forms a hydrogen bond with the side chain of a Glu residue (Glu24 in HmuO and Glu29 in rHO-1 and hHO-1) (25, 28, 29, 36, 44, 45), supporting the plant-type–specific accommodation of heme near the N terminus (31) (Fig. 3*C*).

### Comparison of electrostatic surface potential

A previous spectroscopic analysis suggested that ferric verdoheme, which is the reaction intermediate in heme degradation (Fig. 1*A*), is more stable in plant-type HO than in other HOs (42). We calculated the electrostatic potential of GmHO-1 with the APBS Tool 2.1 plugin (46) in PyMOL (https://pymol.org/2/), and mapped the surface potential with a color gradient from red (negative, −5 kT/e) to blue (positive, 5 kT/e) at pH 7.0 (Fig. 2). Interestingly, the back and side walls of the heme pocket are negatively charged in GmHO-1, whereas the other HOs have positively charged walls (Fig. 2). The difference in the electrostatic surface potential of the heme pocket might explain why verdoheme is more stable in GmHO-1.

We compared the electrostatic potentials of HmuO and hHO-1 mapped on the molecular surface, which showed that the positively charged character of the surface near the bound heme is common to all three HOs, but only GmHO-1 has a dented cave-like surface to accommodate the small globular Fd molecule (Fig. S1). This characteristic of a dented cave–like surface is also found in the molecular surface of Fd–NADP^+^ reductase (FNR; Fig. S1*C*). However, the positively charged area around the heme in GmHO-1 is clearly narrower than that of FNR, implying that it has a weaker interaction with Fd.

### CD, differential scanning calorimetry, ITC, and NMR measurements in solution

**Figure 4:**
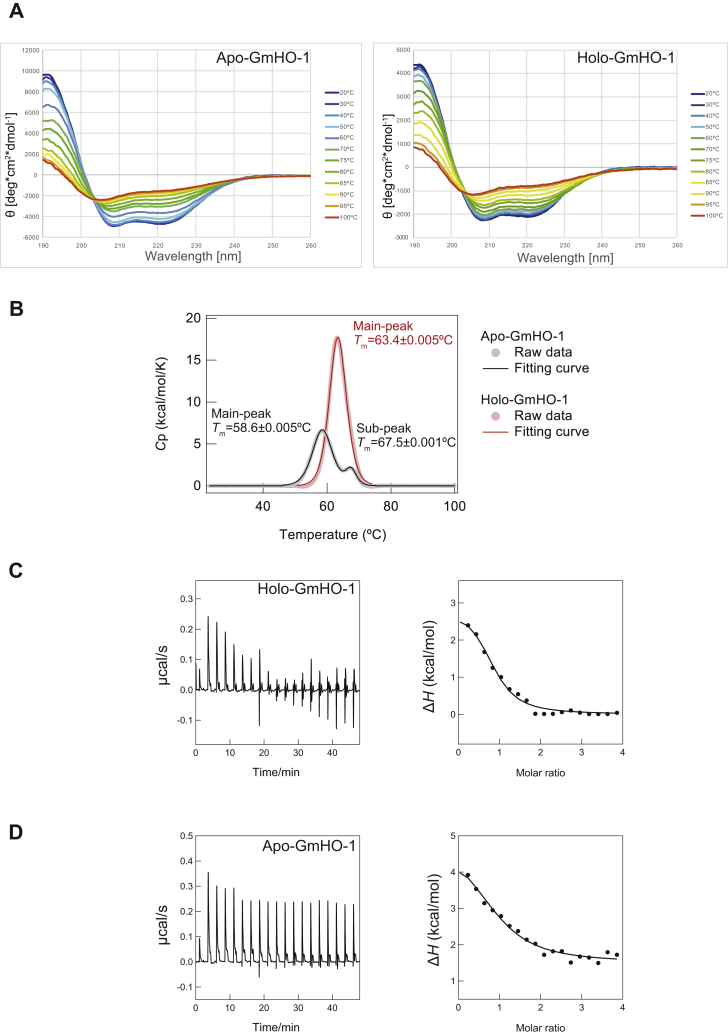
**Solution structure analyses of holo-*Glycine max* (soybean) HO-1 (GmHO-1) and apo-GmHO-1.***A*, temperature-change CD measurements of apo-GmHO-1 and holo-GmHO-1. The spectra show that both GmHO-1 structures contain α-helices, whereas calculations using BeStSel (48, 49) show that the helical content of holo-GmHO-1 is 8.4% less than that of apo-GmHO-1. *B*, DSC charts of apo-GmHO-1 and holo-GmHO-1. *C*, ITC measurements of the interaction of Fd with holo-GmOH-1 and apo-GmHO-1. GmHO-1, *Glycine max* (soybean) HO-1.

The crystallographic analyses of hHO-1 and rHO-1 showed that structural changes occur on heme binding to the apo form (36, 41, 47). We tried to solve the crystal structure of apo-GmHO-1 but failed to obtain crystals. Therefore, to determine structural differences in GmHO-1 with and without heme, we performed CD and differential scanning calorimetry (DSC) measurements on apo-GmHO-1 and holo-GmHO-1 to calculate their helical content and melting temperature (*T*_m_), (Fig. 4, *A*–*B* and Fig. S2*A*).

Although we estimated a difference in helical content of 8.4% between apo-GmHO-1 and holo-GmHO-1 (48, 49), quantitative observation of the small structural changes that occur upon heme binding was difficult because the helical content calculated from the CD spectra was highly dependent on the concentration of protein. The *T*_m_ values of apo-GmHO-1 and holo-GmHO-1 obtained from CD analysis, which reports protein stability based on secondary structures, were almost the same at around 71 °C (Fig. S2*A*).

However, the *T*_m_ values of apo-GmHO-1 and holo-GmHO-1 obtained from DSC analysis, which predominantly reflects the stability of tertiary structures, were 58.6 ± 0.05 (main DSC peak) and 63.4 °C ± 0.07 deg. C, respectively (Fig. 4*B*). A minor peak in the chart of apo-GmHO-1 at 67.5 °C might be related to a further structural change. Thus, we concluded that, as the temperature increased, the 3D structures of apo-GmHO-1 and holo-GmHO-1 first became disordered, and then the secondary structures were melted by heat. Structural differences between the two proteins depending on the presence of heme or noncovalent interactions between heme and apo-GmHO-1 might stabilize the tertiary structure of holo-GmHO-1.

The relatively narrower charged surface in the holo form than in FNR (Fig. S1) raised the question of whether Fd binding in GmHO-1 is heme dependent or not. Next, therefore, we performed a single ITC measurement for Fd binding with the apo form and holo form of GmHO-1 (Fig. 4, *C*–*D*). The heat of reaction indicated that complex formation occurred between the two proteins. ITC analysis indicated that the *n*-value was approximately 1.0 (*i.e.*, one-to-one binding stoichiometry), and there was no allosteric effect of Fd binding in either the apo form or holo form. Δ*H*_bind_ values were positive: +3.3 ± 0.5 kcal/mol for apo-GmHO-1 and +2.6 ± 0.5 kcal/mol for holo-GmHO-1 binding. Positive *T*Δ*S*_bind_ values of Fd binding to the apo form and holo form were also obtained: 9.9 ± 0.5 and 9.8 ± 0.6 kcal/mol, respectively. These results revealed that complex formation between GmHO-1 and Fd is a thermodynamically unfavorable endothermic reaction, and thus driven purely by Δ*S*_bind_. This energetic feature is also observed during formation of the FNR–Fd complex (50). Interestingly, based on *K*_d_ values of Fd binding to apo-GmHO-1 (14.7 ± 4.0 μΜ) and holo-GmHO-1 (5.3 ± 2.2 μΜ), the interprotein affinity between Fd and holo-GmHO-1 is slightly stronger than that between Fd and apo-GmHO-1, thereby implying that Fd binding is partly heme dependent.

To visualize the direct interaction between GmHO-1 and Fd, we tried to crystallize holo-GmHO-1 complexed with Fd. Because we previously used maize Fd (82.6% identity to soybean Fd) for biochemical analysis of GmHO-1 (31, 42) and we confirmed its affinity for GmHO-1 by ITC (Fig. S3), we attempted to crystallize the holo–GmHO-1 complex with both soybean and maize Fd. Unfortunately, however, we did not obtain crystals suitable for X-ray diffraction for either complex. However, several solution NMR analyses of the interaction between maize Fd and Fd-dependent enzymes have been previously reported; therefore, we carried out NMR measurements using maize [^15^N]-Fd (51) as a probe to identify the residue-level interactions between Fd and GmHO-1.

**Figure 5:**
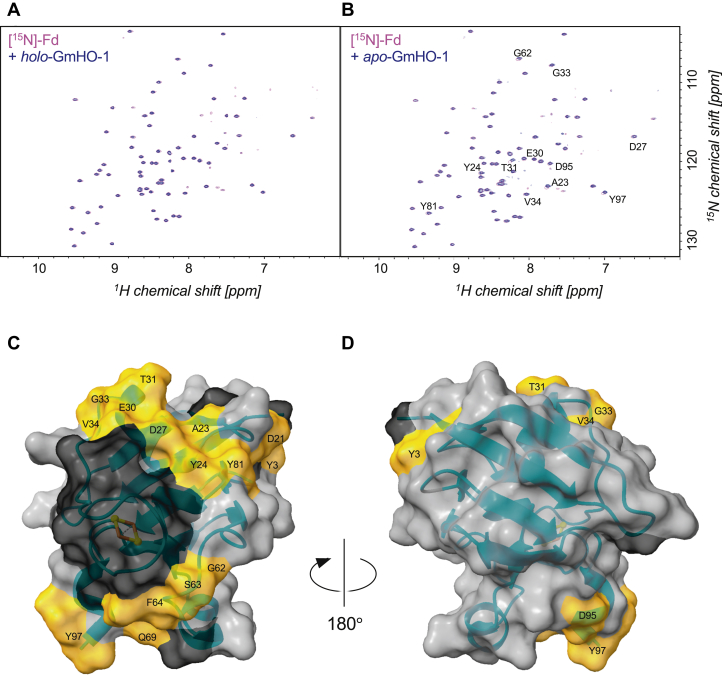
**NMR chemical shift perturbation of [**^**15**^**N]-Fd upon complex formation with holo- and apo-*Glycine max* (soybean) HO-1 (GmHO-1).***A*–*B*, overlays of the 2D ^1^H–^15^N heteronuclear single quantum coherence–transverse relaxation optimized spectroscopy spectra of [^15^N]-Fd are shown in the absence (*magenta*) and presence (*blue*) of 10 molar equivalents of holo-GmHO-1 (*A*) and apo-GmHO-1 (*B*). *C*, location of amino acid residues showing chemical shift perturbation mapped on the surface of the crystal structure of maize Fd III (Protein Data Bank ID: 5h57). *Orange* indicates residues showing perturbation; *black* indicates residues that could not be assigned owing to paramagnetic relaxation of the iron. *D*, view of the molecular surface observed on rotating the image in (*C*) 180° around the *y*-axis. This surface is opposite the iron–sulfur cluster.

We first mixed [^15^N]-labeled native Fd with unlabeled holo-GmHO-1 but observed almost no shift or only broadening in the ^1^H/^15^N peaks derived from the amide groups of Fd (Fig. 5*A*). Assuming that the [2Fe–2S] cluster of Fd and the heme of holo-GmHO-1 are spatially close in the complex, it is possible that fast paramagnetic relaxation because of the irons of [2Fe–2S] and heme inhibits observation of peaks from the contact surface. We therefore prepared apo-GmHO-1 with no heme and conducted a chemical shift perturbation experiment in the same manner. As a result, small but discernible changes in the chemical shifts were observed for the peaks from residues located around the [2Fe–2S] cluster of Fd (Fig. 5, *B*–*C* and Figs. S4 and S5). No such perturbation was observed for residues on the opposite side of the cluster (Fig. 5*D*). This result suggests that the [2Fe–2S] cluster side of Fd interacts with GmHO-1. The rather small changes observed in the chemical shifts may be due to paramagnetic effects hiding the direct interaction sites in the spectrum, as mentioned previously.

We in addition performed a docking simulation with holo-GmHO-1 (this study) and maize Fd (Protein Data Bank [PDB] ID: 5h57) using the HADDOCK server (52) based on NMR chemical shift perturbation experiments of apo-GmHO-1. Ten HADDOCK models were successfully obtained, from which we adopted the top cluster of models with a HADDOCK score of −57.0 ± 2.2 (Fig. S6*A*).

## Discussion

In this study, we have determined the high-resolution crystal structure of GmHO-1 complexed with heme at a resolution of 1.06 Å, which is the highest resolution for an HO structure reported to date. The detailed structure of the heme–GmHO-1 complex provides clues to answer the question why the plant-type HO evolved a unique amino acid sequence with exceptionally low identity to other HOs, including cyanobacterial enzymes. One answer is the insertion of a novel irregularly structured region and the resultant shorter proximal α1 helix (Figs. 1*B* and 3*A*), which leads to an upstream shift of 13 residues for His-ligation.

What is the function of this newly identified irregularly structured region? First, it may be related to the formation of a new tunnel near the heme-binding pocket (Fig. 2), which provides a water channel directly from the active site to the solvent. The enzymatic reaction catalyzed by HO requires a total of eight protons (H^+^) to complete heme degradation and produces two water molecules as byproducts. To facilitate both the release of these water molecules and the uptake of protons to the active site, GmHO-1 might have an alternative direct pathway for the exit of water or supply of protons to or from the bulk solvent. Such an ensured proton/water pathway might act as a device to cope with the dynamic pH change from 7 to 8 that occurs in the chloroplast stroma depending on the fluctuating light conditions around land plants. The flexible region at the proximal helix near the heme pocket also seems to be expedient for product exclusion because the resultant biliverdin is a substrate of the subsequent enzymatic reaction.

Second, the irregularly structured region may function to support the alternative hydrogen-bond network known to be critical in heme–catabolic reactions. In studies of HmuO, the hydrogen-bonding network has been proposed as a pathway for proton transfer from the aqueous cytoplasm to the active site on the distal side of heme (28, 40, 43, 53), where the side chain of Arg132 near heme interacts with Asp136 and fixes the position of the Asp136 side chain to connect the hydrogen-bond network to the water molecules. The importance of Asp136 has been further confirmed in hHO-1 by analysis of an Asp140His mutant (corresponding to Asp136His in HmuO), which showed a significant reduction in activity (43). This raises the question of why the plant-type HO naturally possesses a His residue instead of Asp at this position (31, 42). To understand the natural substitution of these key structural elements, we compared the heme-binding sites of GmHO-1 with those of other HOs (Fig. 3*C*). Although the plant-type HO lacks the conserved Asp and Arg residues for the hydrogen-bonding network observed in hHO-1 and HmuO, it has an alternative hydrogen-bonding network formed by Ala149 and His150. Interestingly, this alternative hydrogen-bonding network in GmHO-1 connects the catalytic active site to the opposite side of the protein surface, differing from the situation in other HOs. In addition, it should be noted that the equivalent hydrogen-bond network was not observed in the structure of apo-hHO-1 (36, 47). The hydrogen-bonding network on the distal side that is essential for enzymatic activity is probably induced by heme binding to GmHO-1.

It is unclear whether the structures of the apo form and holo form of GmHO-1 are different because we failed to obtain apo form crystals and could not perform structural analyses. Presumably, the thermal instability of apo-GmHO-1 relative to holo-GmHO-1 hindered crystallization of the GmHO-1 protein, as implied by the 5 °C increase in *T*_m_ value observed by DSC upon heme binding to GmHO-1. In addition, some hydrophobic and hydrophilic interactions were found between heme and the N-terminal residues of GmHO-1 (Arg23, Met27, His30, Gln34; calculated by Ligplot+ (54); Fig. 3*A*). Without a bound heme molecule, the N-terminal regions might be structurally unstable and possibly even nonstructured. A similar unstructured zone has also been found in the N-terminal region including the proximal helix of rHO-1 (∼30 amino acid residues) (47). The existence of an unstructured N-terminal region is also supported by our ITC measurements of the interaction of GmHO-1 with Fd, based on the *K*_d_ values for apo-GmHO-1 and holo-GmHO-1 (14.7 and 5.3 μΜ). In other words, the affinity of Fd seems to be higher for holo-GmHO-1 than for apo-GmHO-1.

Third, it is possible that the new structural features furnished in GmHO-1 have emerged in plant-type HOs as part of evolutionary tuning for Fd dependency. Data from the ITC and NMR measurements revealed that both apo-GmHO-1 and holo-GmHO-1 have affinity for Fd. This intriguing feature is common to other Fd-dependent enzymes; for example, FNR can bind Fd even without bound NADP^+^. Another interesting coincidence between GmHO-1 and other Fd-dependent proteins relates to the missing electron density for the N-terminal 15 residues in GmHO-1, which is likely to be due to the intrinsic flexibility of the N terminus. This flexible region also contains three positively charged lysine residues. Flexible N-terminal regions containing basic residues are common to other Fd-dependent proteins, including FNR, sulfite reductase, and NdhS (Fd-binding subunit of photosynthetic complex I: NDH-1L) (51, 55, 56, 57). The aspect of the electrostatic surface potential of GmHO-1 is also consistent with the idea of evolutionary tuning for Fd dependency. The positively charged character of the molecular surface near the bound heme is present in the structure of all three HOs (so far determined), but only GmHO-1 has a dented cave–like surface suitable for accommodating the small globular Fd protein.

Our ITC and NMR measurements revealed that the binding of Fd to GmHO-1 is entropy driven and that Fd contacts the enzyme at the [2Fe–2S] site to face the heme-binding site of GmHO-1, in a rational manner. The predicted GmHO-1:Fd models derived from HADDOCK calculation support this docking model of Fd (Fig. S6*A*). The potential path of electron transfer between GmHO-1 and the modeled Fd is thought to occur by direct electron transfer through space because the closest distance between the heme of GmHO-1 and the [2Fe–2S] cluster of Fd is about 5 Å (the distance between the CBA carbon in heme and the S2 sulfur atom in the [2Fe–2S] cluster ranges from 5.1 to 5.5 Å).

In the case of mammalian HO, the structure of the electron transfer complex formed between HO and CPR has been revealed by X-ray crystallography (23) (Fig. S6*B*). Because the CPR molecule is much bigger than plant-type Fd, the interface between rHO-1 and CPR is much wider and constituted by not only electrostatic but also the other types of interaction. The distance between heme and FMN in that complex (6 Å) also implies direct electron transfer from FMN to heme, which thus seems to be common to both types of HOs.

The electrostatic surface potential of GmHO-1 seems to be suitable to stabilize the short-lived intermediate, verdoheme, in plant-type HOs. During the multistep HO reactions, GmHO-1 consumes seven electrons to complete the entire reaction, which means that Fd sequentially contacts the active site seven times. In particular, in the conversion step from verdoheme to ferric biliverdin, three electrons are required at the same time. As mentioned in the Results section, the inside of the heme pocket of GmHO-1 is negatively charged, unlike those of other HOs, being complementary to the positive charge on verdoheme. We propose that the electrostatic interactions between verdoheme and protein might stabilize the intermediate in the heme pocket, thereby ensuring the acceptance of electrons from Fd. In chloroplast stroma, where the plant HOs function, many other Fd-dependent enzymes compete with each other to recruit reduced Fd molecules, and the redox level of the thylakoid membrane of higher plants changes dynamically because of fluctuating light conditions. Thus, the plant-type HO might need more capability to optimize Fd binding and stabilize substrate binding as compared with other HOs.

In summary, we have determined the X-ray structure of GmHO-1 with bound heme at a resolution of 1.06 Å, which is the highest resolution among the currently available HO structures, and conducted ITC and NMR chemical shift perturbation studies of Fd binding. Our combination of X-ray crystallography, ITC, and NMR data has helped to elucidate two specific features of the GmHO-1 structure. The binding site of heme in GmHO-1 is different from that in other types of HO to some degree; yet catalytically essential elements, such as the hydrogen-bonding network and His ligation, are structurally conserved. The newly identified water channel near the heme pocket and the unique surface structure including distribution of the electrostatic potential strongly suggest that the evolutionary optimization has adapted GmHO-1 for the physiological demands specific to plant-type HOs. To confirm our proposed interpretations of the structural optimization unique to the plant-type HO, more functional studies will be needed.

## Experimental procedures

### Expression and purification of native GmHO-1 and heme–GmHO-1

The expression plasmid for GmHO-1 was prepared as described previously (31) and transformed into *Escherichia coli* strain B834 (DE3) pLysS. Cells were precultured at 37 °C overnight and transferred to 6 L of LB medium. When the optical density at 600 nm reached 0.8 to 1.0, the incubation temperature was decreased to 25 °C, and the cells were continuously cultured for 24 h. The cells were harvested by centrifugation at 4500*g* for 5 min using a JLA-9.1000 rotor (Beckman Coulter Co, Ltd) and stored at −20 °C. Frozen cells were resuspended in 90 ml of lysis buffer (50 mM Tris–HCl (pH 7.5) and 2 mM EDTA) and disrupted by sonication using a BRANSON 450 Sonifier. After sonication, the lysate was clarified by ultracentrifugation at 100,000*g* for 1 h using a HITACHI P45AT rotor. The supernatant was applied to a DE-52 column (2.6 × 28 cm) equilibrated with lysis buffer. The column was washed with 5 column volumes of buffer A (20 mM K–Pi buffer [pH 7.4]) and eluted by buffer B (20 mM K–Pi [pH 7.4] and 50 mM KCl). The eluted sample was dialyzed against 2 l of buffer A for 16 h at 4 °C. The sample was then applied to a HiTrap Q HP column (GE Healthcare), which was washed with buffer A. The protein sample was eluted with 500 ml of buffer C (20 mM K–Pi buffer [pH 7.4] and 250 mM KCl), using a linear gradient of 0 to 250 mM KCl on an AKTA prime system. The elution profile was monitored at 280 nm, and the main peak containing the crude sample of apo-GmHO-1 was fractionated. The fractionated sample was applied to a Phenyl Sepharose column (GE Healthcare) equilibrated with buffer D (20 mM K–Pi buffer [pH 7.4] and 50% saturated ammonium sulfate) and eluted with buffer A using a linear gradient of 50 to 0% saturated ammonium sulfate and an AKTA prime system. Before applying the collected sample to a HiLoad 16/600 Superdex 75 column (GE Healthcare), it was concentrated to less than 5 ml with an Amicon Ultra-15 unit (10 kDa; Merck Millipore) and centrifugation (4000*g* at 4 °C). The concentrated sample was filtered through a Millex-GV membrane (0.22 μm, polyvinylidene fluoride) and loaded onto a Hiload 16/600 Superdex 75 column equilibrated with buffer A. The purified sample showed a single band of 26 kDa on SDS–PAGE (Fig. S7*A*). The purified apo-GmHO-1 was concentrated by an Amicon Ultra-15 unit. Heme–GmHO-1 was prepared as described previously (31).

### Purification of Fd and its NMR samples

The expression plasmids for soybean and maize Fd type III were prepared as described previously (55, 58). Native soybean and maize Fd proteins were expressed in *E. coli* strain BL21 (DE3) using LB medium (100 μg/ml ampicillin), whereas [^15^N]-labeled and [^15^N, ^13^C]-labeled maize Fd proteins were expressed in *E. coli* strain BL21 (DE3) using M9 medium containing either 1 g/l of ^15^NH_4_Cl or 1 g/l of ^15^NH_4_Cl and 4 g/l of [^13^C]-glucose, respectively. The transformed *E. coli* cells were grown overnight in 20 ml of LB medium (100 μg/ml ampicillin) at 37 °C and scaled up to 6 l of LB medium. When the optical density at 600 nm reached 0.4 to 0.5, IPTG was added to a final concentration of 0.1 mM, and the cells were cultured for 24 h at 25 °C. Cells were harvested by centrifugation at 4500*g* for 5 min using a JLA-9.1000 rotor (Beckman Coulter Co, Ltd) and stored at −20 °C. The frozen *E. coli* cells were resuspended in 90 ml of lysis buffer (50 mM Tris–HCl [pH 7.5] and 50 mM NaCl) and were disrupted by sonication. The lysate was then clarified by ultracentrifugation at 120,000*g* for 30 min at 4 °C using a HITACHI P45AT rotor. The soluble fraction was applied to a DE-52 column (2.6 × 28 cm), and the column was washed with wash buffer (50 mM Tris–HCl [pH 7.5] and 100 mM NaCl) and eluted by elution buffer (50 mM Tris–HCl [pH 7.5] and 500 mM NaCl). The eluted fraction was dialyzed against 2 l of 50 mM Tris–HCl (pH 7.5) for 16 h at 4 °C. The sample was then applied consecutively to Hitrap Q HP (GE Healthcare), Phenyl Sepharose (GE Healthcare), and Hiload 16/600 Superdex 75 (GE Healthcare) columns. The purified sample showed a single band of 10 kDa on SDS–PAGE (Fig. S7*B*). The purified sample was concentrated by using an Amicon Ultra-15 unit. The protein concentration of native Fd was calculated by using its molar extinction coefficient (*ε*_420_ = 9.68 mM^−1^ cm^−1^).

### Crystallization of heme–GmHO-1

Heme–GmHO-1 crystals were obtained by the hanging-drop vapor diffusion method at 277 K. The hanging drop was prepared by mixing equal volumes of protein solution (30 mg/ml) and reservoir solution containing 60 mM citric acid, 40 mM Bis–Tris propane (pH 4.1), 16% (w/v) PEG 3350 and 3.2% (v/v) dimethyl sulfoxide. For X-ray intensity data collection, single crystals were transferred to a cryo-protectant solution containing the same crystallization buffer with 35% (w/v) PEG 3350 and dipped into liquid nitrogen.

### Crystallographic data collection and structural determination

Native data were collected on beamline BL44XU at SPring-8 using an EIGER X 16M detector (Dectris) at cryogenic temperature (100 K). The Fe–SAD data were collected on beamline TPS 05A at the National Synchrotron Radiation Research Center using a CCD detector MX-300HE (Rayonix) at cryogenic temperature (100 K). The native data set was processed and scaled by using XDS (59), and diffraction data with 98.3% completeness were obtained at a resolution of 1.06 Å from 1500 frames. The SAD data set was processed and scaled by using HKL2000 (60), and diffraction data with 90.9% completeness were obtained at a resolution of 1.84 Å from 360 frames. The crystals of heme–GmHO-1 belonged to space group *P*2_1_ with cell dimensions of *a* = 37.7 Å, *b* = 45.6 Å, *c* = 72.4 Å, *β* = 101.1°, and contained one molecule in the asymmetric unit. The initial phase was determined by the SAD method using Phenix Autosol (61); the phase was then extended to a resolution of 1.06 Å. Structural refinement was performed by using phenix.refine (62), SHELXL (33), and COOT (34). The data collection and refinement statistics are shown in Table 1.

### ITC measurement

All ITC measurements were performed with a MicroCal PEAQ-ITC instrument (Malvern Panalytical, UK) at 25 ^o^C. The concentrations of Fd in the syringe and GmHO-1 in the cell were 800 and 40 μM, respectively. All protein solutions were subjected to buffer exchange into 20 mM Tris–HCl buffer (pH 7.0) containing 20 mM NaCl using PD-10 columns (GE Healthcare Life Sciences), and air bubbles were removed by centrifugation for 5 min at 10,000*g* prior to ITC. The following ITC parameters were used: titration, 19 injections; initial delay, 60 s; spacing time, 150 s; reference power, 10 μcal; and stirring speed, 500 rpm. The injection volume was 0.4 μl for the first injection and 2 μl for the remaining injections. The heat of dilution was measured by titrating 800 μM Fd in the syringe into a sample cell filled with buffer alone. The heat flow and binding isotherm were calculated by subtracting the heat of dilution. The data were fitted to the one set of sites-binding model in MicroCal PEAQ-ITC analysis software.

### NMR measurement

NMR experiments were conducted on BrukerBioSpin Avance III spectrometers using TCI triple-resonance cryogenic probes with ^1^H resonance frequencies of 500 and 800 MHz. 2D ^1^H–^15^N heteronuclear single-quantum correlation, 3D HNCACB, CBCA(CO)NH, HNCA, HN(CO)CA, HNCO, and HN(CA)CO spectra (63) were acquired for assignment of the backbone signals of 0.5 mM [^15^N, ^13^C]-labeled maize Fd III dissolved in 50 mM potassium phosphate buffer (pH 7.0) containing 10 mM KCl and 10% (v/v) deuterium oxide for lock at 298 K on a 500 MHz spectrometer (Fig. S8). Chemical shift perturbation experiments were performed by recording 2D ^1^H–^15^N transverse relaxation optimized spectroscopy heteronuclear single-quantum correlation spectra of 50 μM [^15^N]-labeled maize Fd III dissolved in the aforementioned buffer in the presence and absence of 500 μM nonlabeled holo-GmHO-1 and apo-GmHO-1 at 298 K on the 800 MHz spectrometer. The spectra were measured with ^1^H and ^15^N acquisition periods of 71 and 53 ms, respectively. The experimental time for each spectrum was 3 h with 24 scans accumulated for each FID. All NMR data were processed by NMRPipe (64), and spectra were analyzed with NMRFAM-Sparky (65) and MagRO (66). The simulated docking models were calculated by HADDOC 2.4 through submitting the coordinates to the HADDOCK web server (https://wenmr.science.uu.nl/haddock2.4/). Interacting residues were set by the criteria as same as the colored residues in Figure 5, *C*–*D* together with the heme molecule of GmHO-1.

### CD spectra measurement

CD spectra were measured in a 0.1 cm quartz cell from 190 to 250 nm using the continuous-scan option (20 nm/min), with a step size of 0.1 nm and a bandwidth of 1 nm by a J-1500 spectropolarimeter (JASCO) at 20 to 100 °C under constant nitrogen flux. Samples of 0.1 mg/ml apo-GmHO-1 and 0.1 mg/ml heme–GmHO-1 were prepared in 20 mM K–Pi (pH 7.4) buffer. The relative helical content was calculated by BeStSel (48, 49).

### DSC measurement

DSC measurement was performed on a MicroCal PEAQ-DSC System (Malvern Panalytical). Each sample was prepared at 1.0 mg/ml in 20 mM K–Pi buffer (pH 7.4). The temperature range was set as 20 to 100 °C, and the scan rate was 60 °C/h. The data were analyzed using the MicroCal PEAQ-DSC software.

### Data availability

The coordinates and structure factors for GmHO-1 have been deposited in the worldwide PDB at PDB Japan under accession number 7CKA. The resonance assignment for Maize Fd has been deposited in the Biological Magnetic Resonance Bank as accession number BMR26301. The deposited PDB and Biological Magnetic Resonance Bank data sets are available from the wwPDB and BMRB Web sites upon publication, and the other data sets during and/or analyzed during the present study available from the corresponding author on reasonable request.

## Conflict of interest

The authors declare that they have no conflicts of interest with the contents of this article.
