# Supplementary material for: Crystal structure of higher plant heme oxygenase-1 and its mechanism of interaction with ferredoxin
Source: J Biol Chem. 2020 Dec 24;296:100217. doi: 10.1074/jbc.RA120.016271 (PMC7948506; doi:10.1074/jbc.RA120.016271)
Supplement: Figures S1–S8 [file mmc1.pdf]

# Supporting Information

## Crystal structure of higher plant heme oxygenase-1 and its mechanism of interaction with ferredoxin

**Rei Tohda[1,2], Hideaki Tanaka[1,2], Risa Mutoh[1#], Xuhong Zhang[3], Young-Ho Lee[4,5,6,7], Tsuyoshi Konuma[8], Takahisa Ikegami[8], Catharina T. Migita[9], Genji Kurisu[1,2]**

[1]Institute for Protein Research, Osaka University, Suita, Osaka 565-0871, Japan
[2]Department of Macromolecular Science, Osaka University, Toyonaka, Osaka 560-0043, Japan.
[3]Graduate School of Medical Science, Yamagata University, Yamagata, Yamagata 990-9585, Japan
[4]Research Center of Bioconvergence Analysis, Korea Basic Science Institute, Ochang, Cheongju, Chungbuk 28119, South Korea
[5]Graduate School of Analytical Science and Technology, Chungnam National University, 99 Daehak-ro, Yuseong-gu, Daejeon 34134, South Korea
[6]Neurovascular Research Group, Korea Brain Research Institute, 61, Cheomdan-ro, Dong-gu, Daegu 41062, South Korea
[7]Bio-Analytical Science, University of Science and Technology, Gajeong-ro, Yuseong-gu, Daejeon 34113, South Korea
[8]Graduate School of Medical Life Science, Yokohama City University, Tsurumi-ku, Yokohama 230-0045, Japan
[9]Department of Biological Chemistry, Yamaguchi University, Yoshida Yamaguchi 753-8515, Japan.

[#]Present address: Faculty of Science, Fukuoka University, Jonan, Fukuoka 814-0180, Japan

[*]To whom correspondence should be addressed: Genji Kurisu: Institute for Protein Research, Osaka University, Suita Osaka 565-0871, Japan; gkurisu@protein.osaka-u.ac.jp

**Figure S1.** Comparison of the electrostatic potential of various HO-1s, Fd, and FNR.

**Figure S2.** Melting temperature calculation.

**Figure S3.** ITC measurements of the interaction of maize Fd with holo-GmOH-1 and apo-GmHO-1

**Figure S4.** NMR chemical shift perturbation of Fd upon the interaction with apo-GmHO-1.

**Figure S5.** Changes in the NMR chemical shifts and peak heights of Fd upon the interaction with apo-GmHO-1.

**Figure S6.** HADDOCK model of GmHO-1 and Fd.

**Figure S7.** SDS-PAGE and gel-filtration chart of purified GmHO-1 and Fd.

**Figure S8.** Assignment of the amide groups of maize Fd.

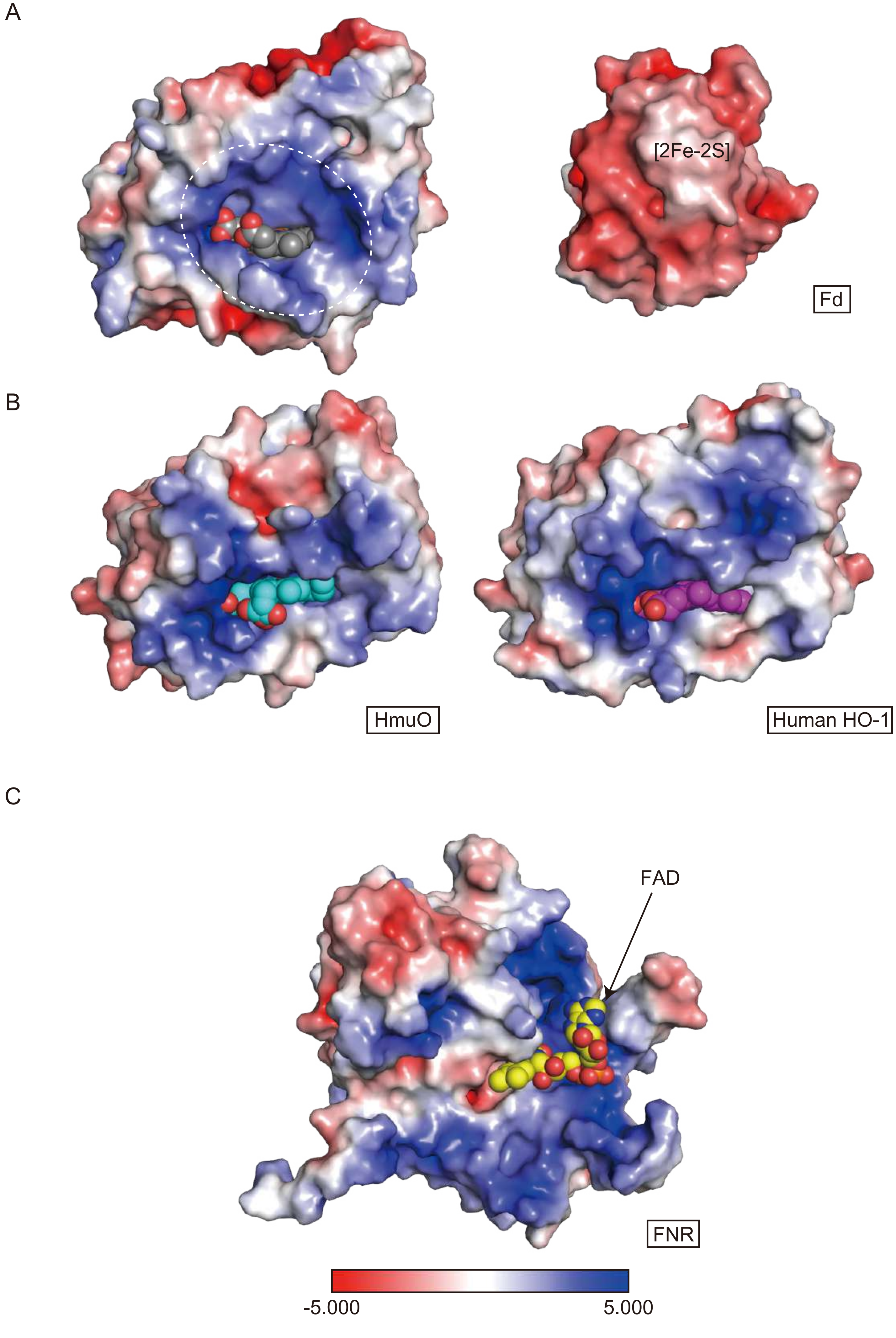
A
[2Fe-2S]
Fd
B
HmuO
Human HO-1
C
FAD
FNR
-5.000
5.000

**Supplementary Figure 1.** Comparison of the electrostatic potential of various HO-1s, Fd, and FNR. A, Electrostatic potential of heme–GmHO-1 and Fd. Fd contains a [2Fe-2S] cluster, and electrostatic potential around the [2Fe-2S] cluster is negatively charged (52). This suggests that GmHO-1 may assemble with Fd by electrostatic interactions between its positively charged area around heme and the negatively charged area of Fd. Heme is shown as a CPK model. B, Electrostatic potential in HmuO and hHO-1. In each HO-1, the electrostatic potential surrounding heme, including the inside of the heme pocket, is positively charged. This shows that the environment of heme differs considerably between GmHO-1 and other HO-1s. C, Electrostatic potential of FNR. The flavin adenine dinucleotide (FAD) domain is the Fd recognition site and the electrostatic potential around FAD is positively charged. FAD is shown as a CPK model in yellow.

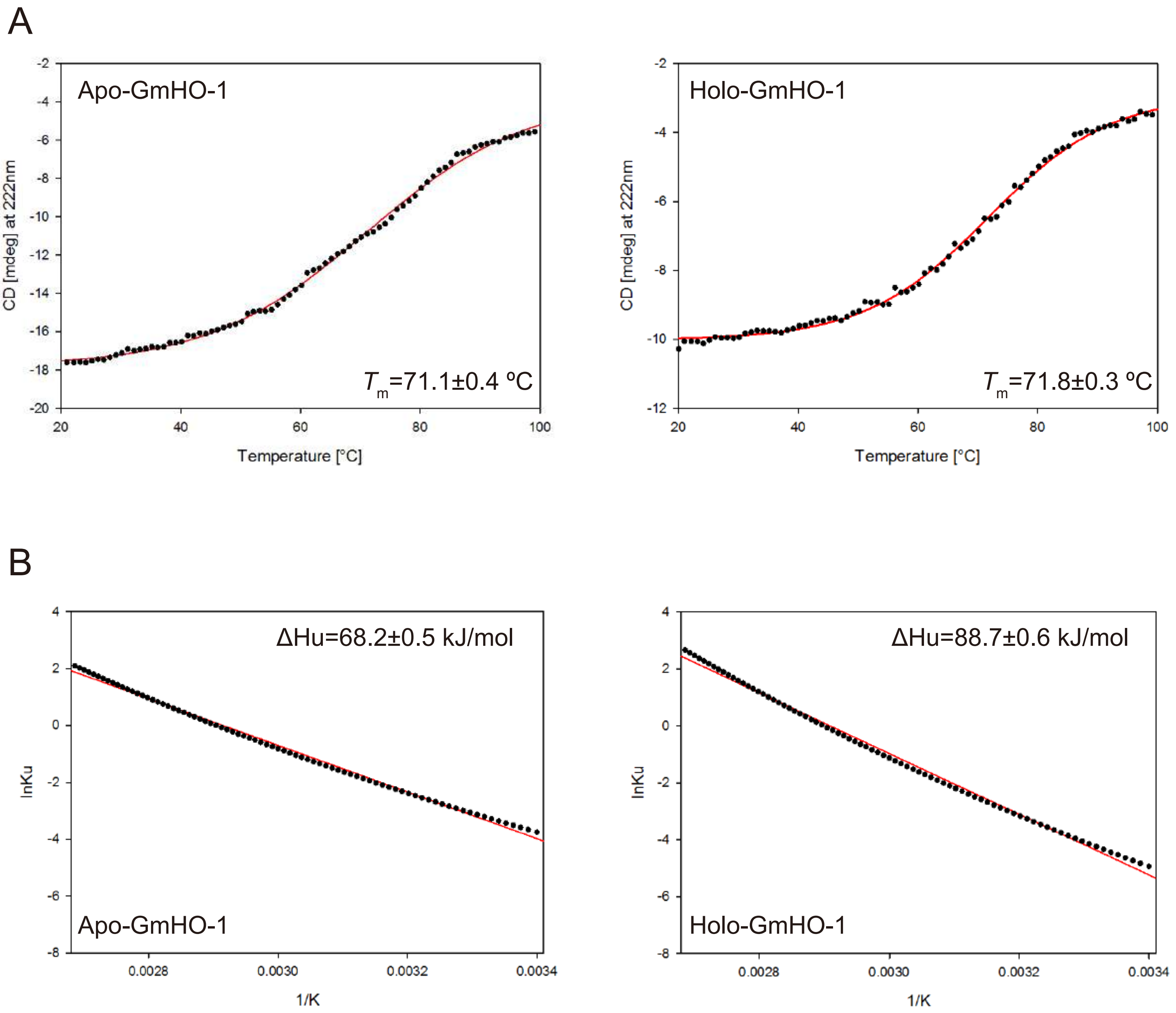


**Supplementary Figure 2.** Melting temperature calculation. A, Change in molar ellipticity of apo- and holo-GmHO-1 at 222 nm. B, Van't Hoff plots of the data in (A) as a two-state transition.

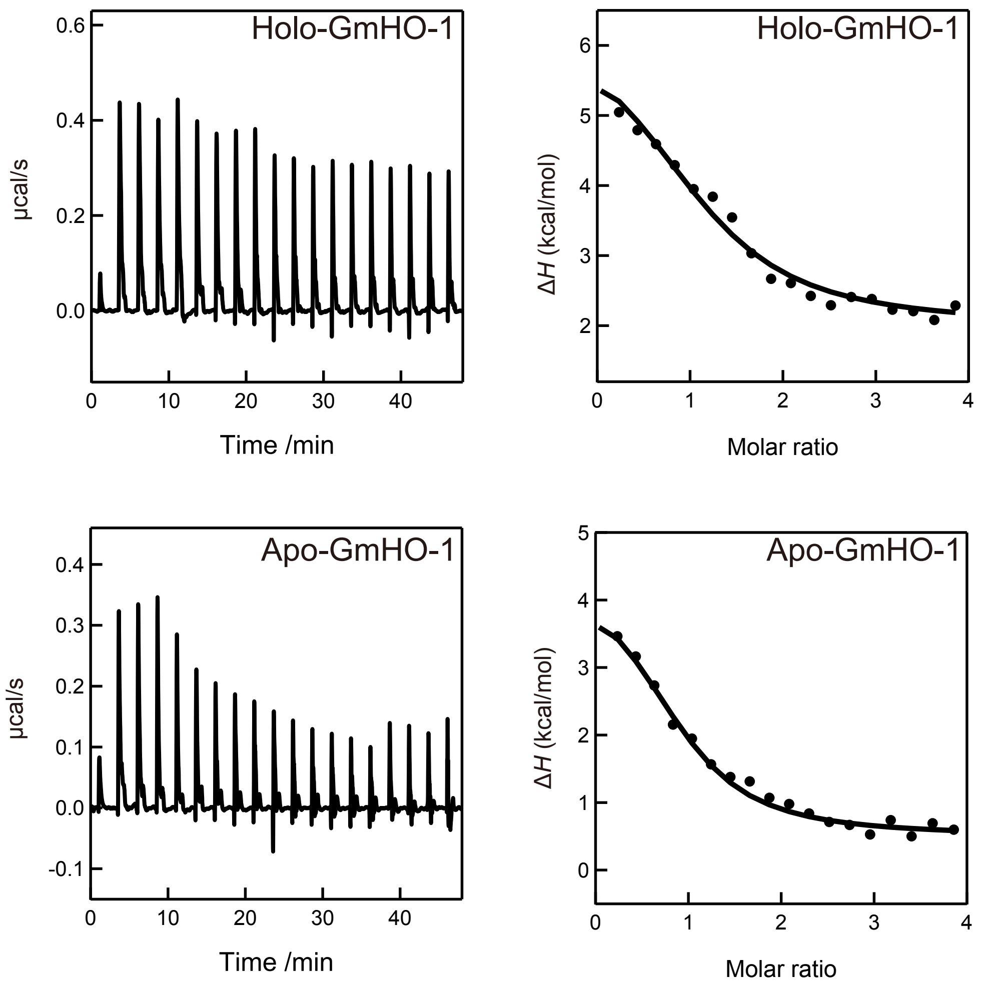


**Supplementary Figure 3.** ITC measurements of the interaction of maize Fd with holo-GmOH-1 and apo-GmHO-1.

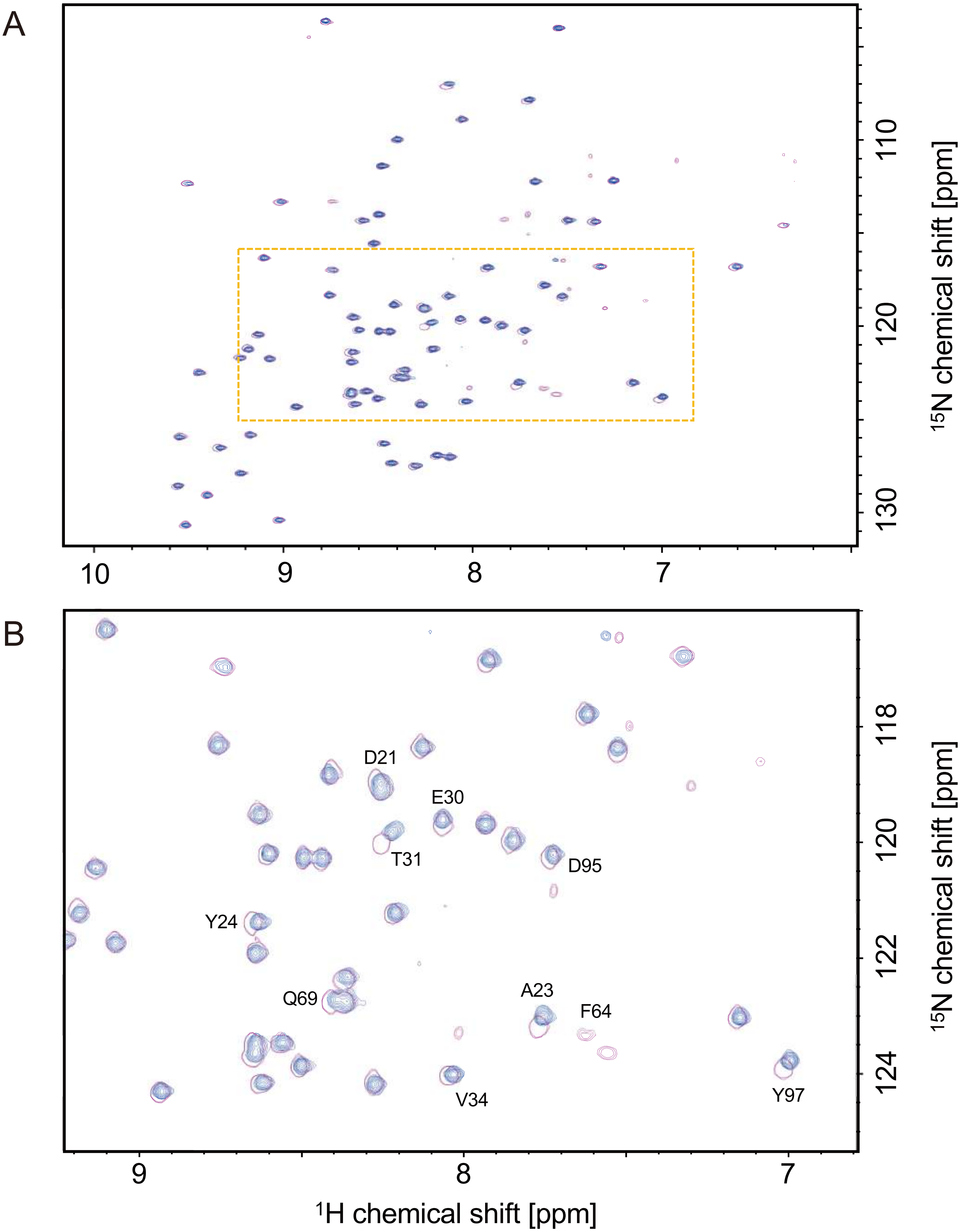


**Supplementary Figure 4.** NMR chemical shift perturbation of Fd upon the interaction with apo-GmHO-1. A, Overlay of the 2D $^{1}$H-$^{15}$N HSQC-TROSY NMR spectra of 50 μM maize [$^{15}$N]-Fd in the absence (magenta) of 500 μM of apo-GmHO-1 and that in the presence (blue) of 500 μM of apo-GmHO-1 (same images as Fig. 5B). B, Enlargement of the boxed area in (A). Peaks that showing chemical shift changes larger than 0.014 ppm upon complex formation are labeled by the corresponding amino acid (also shown in orange in Figure 5 C and D).

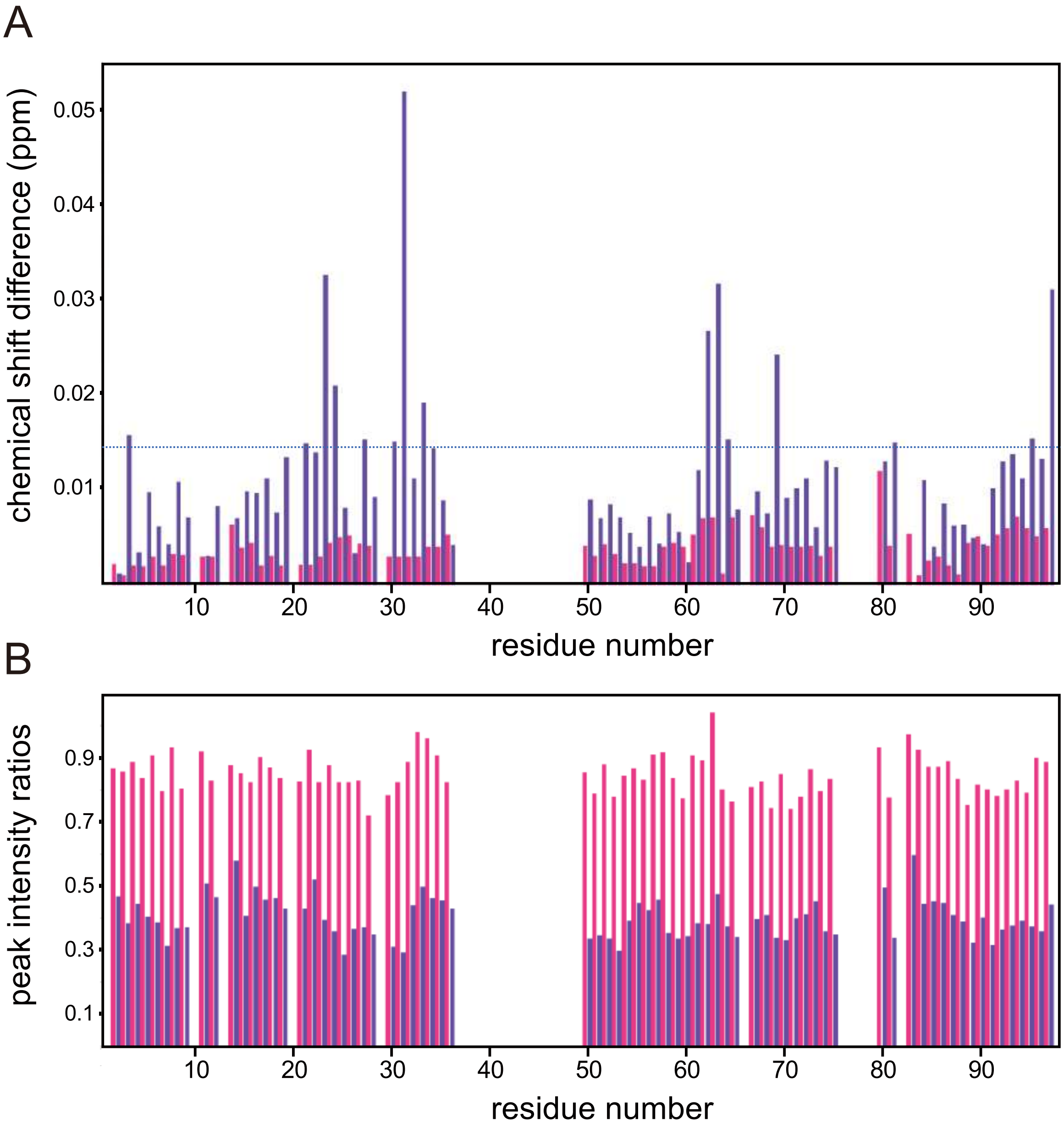


**Supplementary Figure 5.** Changes in the NMR chemical shifts and peak heights of Fd upon the interaction with apo-GmHO-1. A, Changes in chemical shift (CS) observed upon the addition of 500 μM apo- (blue) or holo- (magenta) GmHO-1 to 50 μM [$^{15}$N]-maize Fd plotted against the residue number of Fd. The CS differences, $\Delta\delta^{1}$H and $\Delta\delta^{15}$N for $^{1}$H and $^{15}$N, respectively, were averaged according to the equation $\{(\Delta\delta^{1}\mathrm{H})^2 + (0.17*\Delta\delta^{15}\mathrm{N})^2\}^{1/2}$, where 0.17 is a weighting factor so that the scale of the perturbation of $^{15}$N CS is corrected to that of $^{1}$H CS. Residues with perturbations above 0.014 ppm (horizontal dotted line) in CS are colored orange in Figure 5 C and D. The standard deviation of errors was estimated to be 0.001 ppm from a comparison of peaks that were not perturbed. B, Ratios of peak heights plotted in the same style as panel (A). Standard deviations of errors were estimated from the noise levels of the spectra to be 0.021 and 0.017 for the complex formation with apo- and holo-GmHO-1, respectively.

A

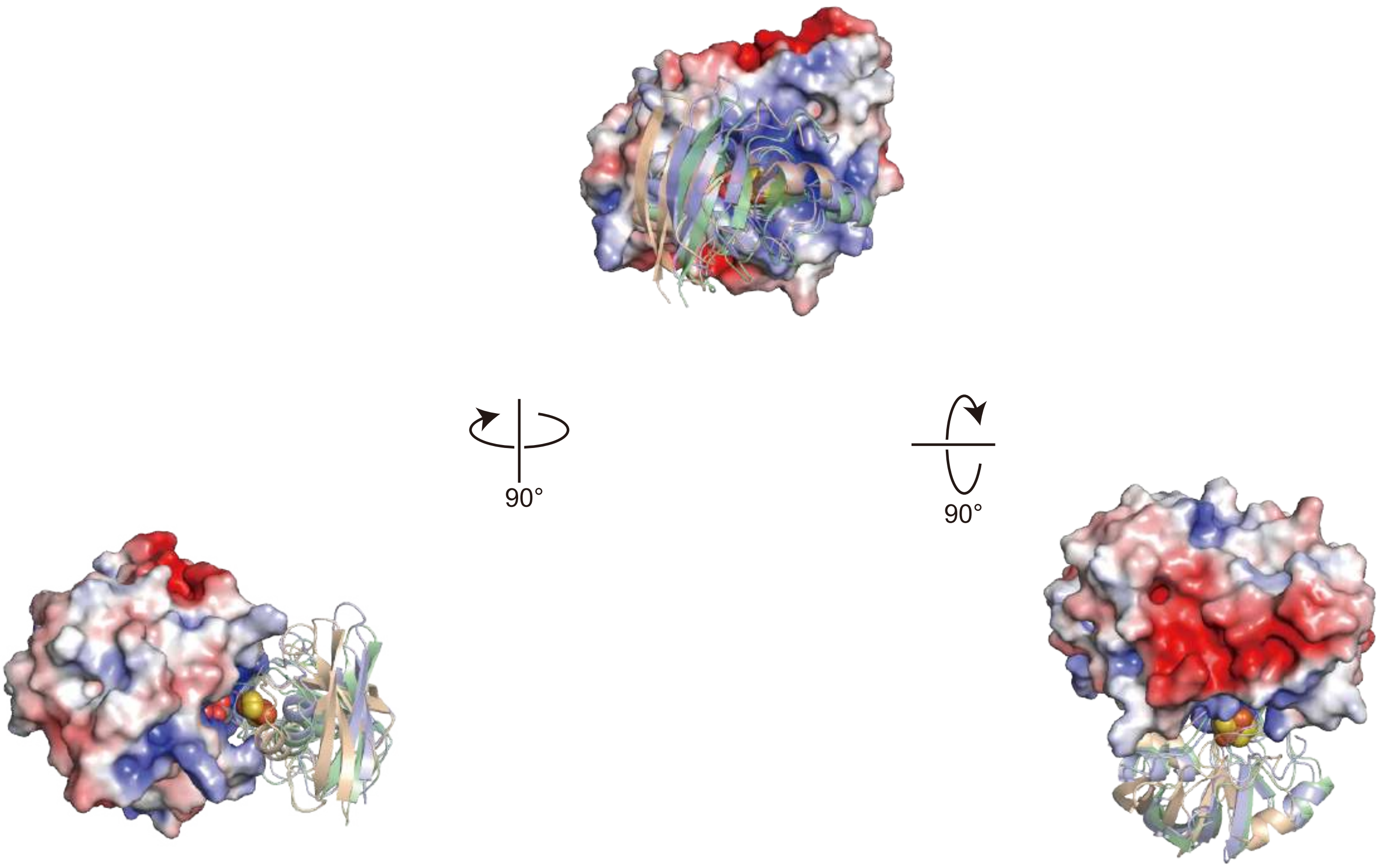


B

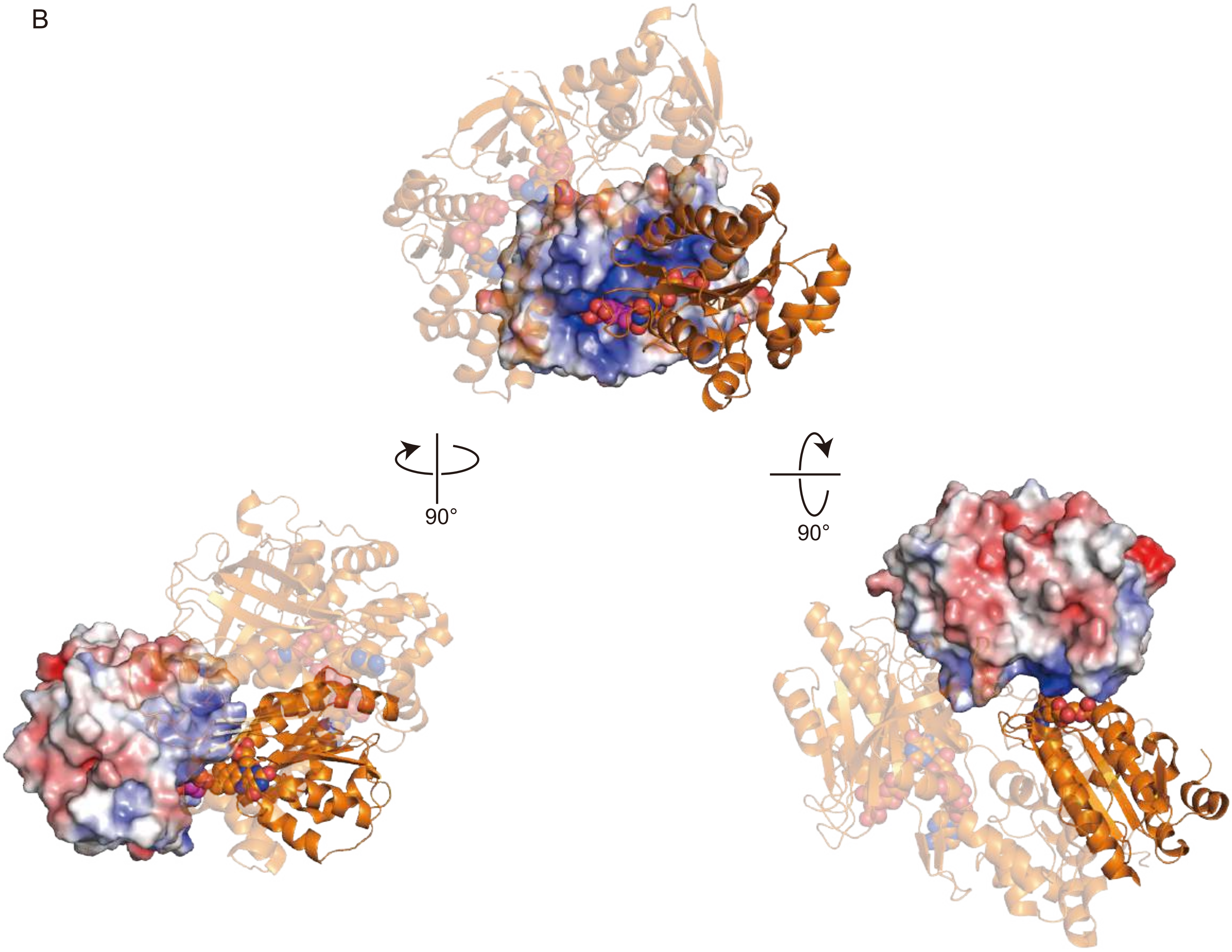

**Supplementary Figure 6.** HADDOCK model of GmHO-1 and Fd. A, Electrostatic potential mapped on the molecular surface of GmHO-1 with the top three models of Fd superimposed (green; 1st, pale orange; 2nd, light blue; 3rd). B, Crystal structure of rHO-1 shown as a molecular surface colored with electrostatic potential and rat CPR as an orange cartoon with prosthetic groups as CPK models.

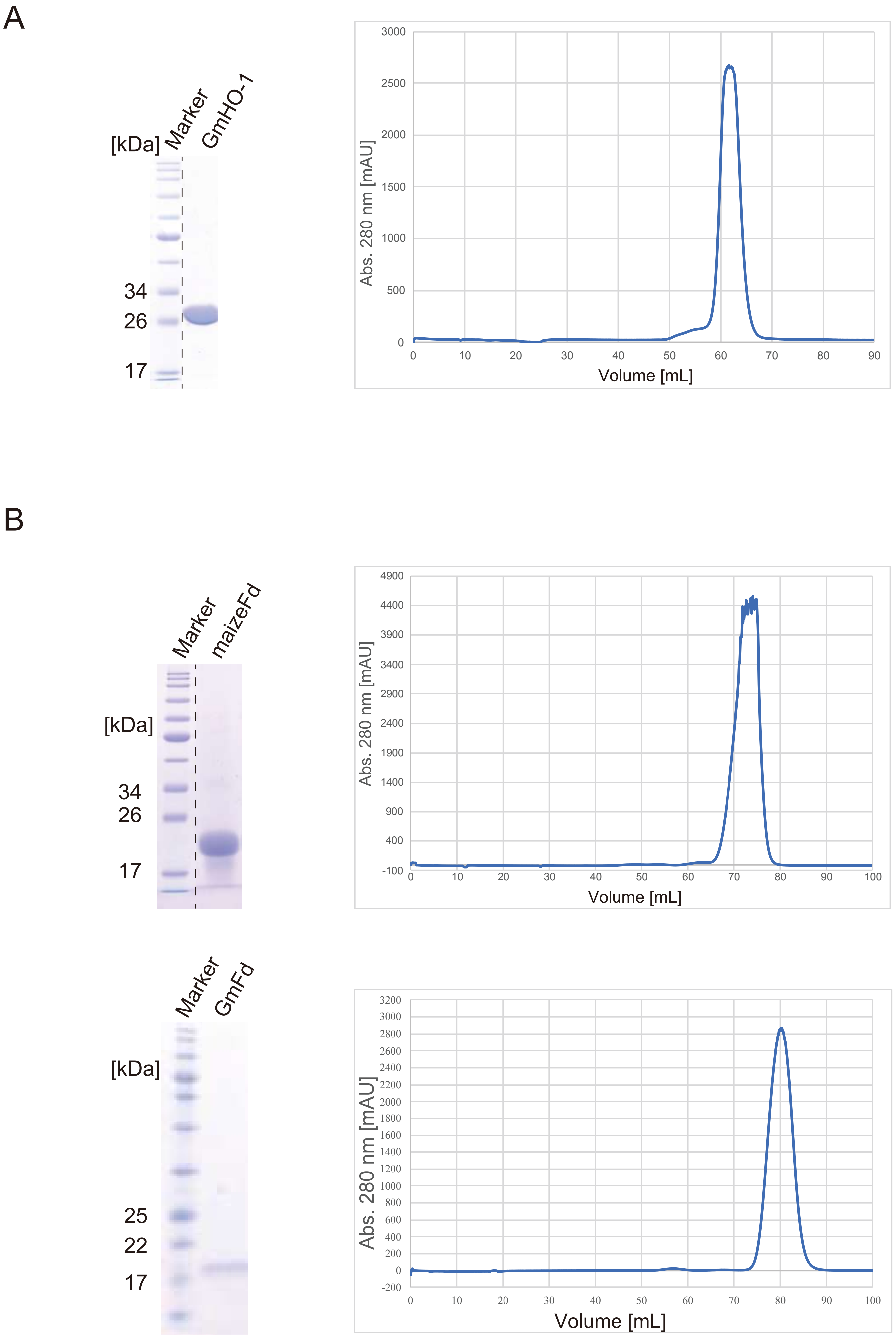


**Supplementary Figure 7.** SDS-PAGE and gel-filtration chart of purified GmHO-1 and Fd. A, GmHO-1. B, Maize (top) and soybean (bottom) Fd.

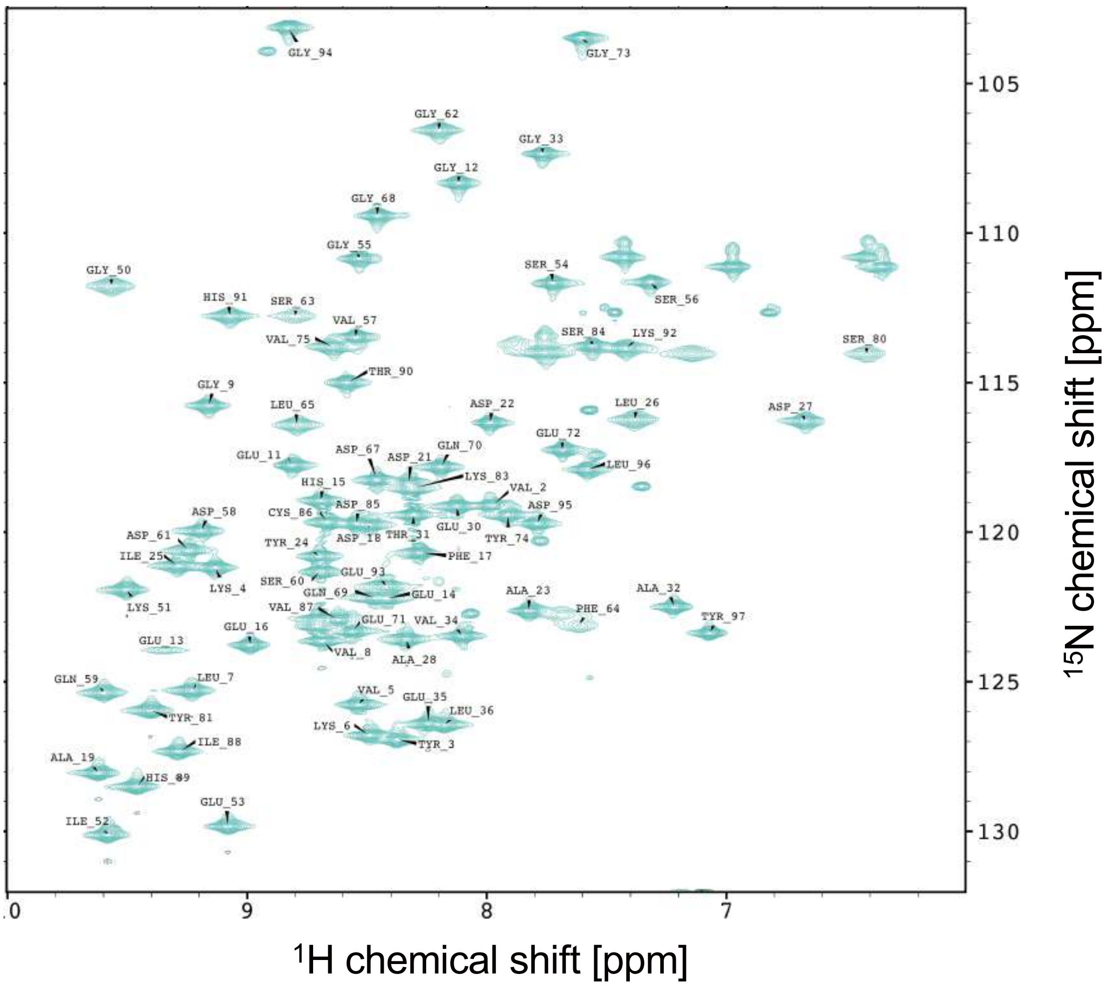


**Supplementary Figure 8.** Assignment of the amide groups of maize Fd. A 2D $^{1}H$-$^{15}N$ HSQC-TROSY NMR spectrum of maize Fd. Amide groups of the main chain were assigned by a series of 3D spectra. Each peak is labeled with the corresponding assigned amino acid.
